# Topic Editorial on Fiber-Optic Sensors

**DOI:** 10.3390/mi15121452

**Published:** 2024-11-29

**Authors:** Muhammad A. Butt

**Affiliations:** Institute of Microelectronics and Optoelectronics, Warsaw University of Technology, Koszykowa 75, 00-662 Warsaw, Poland; ali.butt@pw.edu.pl

Fiber-optic sensors are highly significant in modern technology due to their unique abilities and versatility [1,2,3]. These sensors utilize the transmission of light through optical fibers to detect and measure various physical, chemical, or environmental changes such as temperature, pressure, strain, or even chemical composition [2,4,5]. Their lightweight and compact nature makes them ideal for applications in which traditional sensors are impractical [6]. Fiber-optic sensors are immune to electromagnetic interference, making them indispensable in environments with high levels of electrical noise, such as power plants or military operations [7,8]. Additionally, they can operate in harsh conditions, including extreme temperatures and corrosive environments, due to the robust nature of the optical fibers. These sensors are pivotal in industries such as telecommunications, structural health monitoring, aerospace, and medical diagnostics, offering high sensitivity, long-range measurement capabilities, and real-time data transmission [9]. Their ability to support distributed sensing over vast areas, for instance in pipeline monitoring or earthquake detection, further underscores their importance in advancing the safety and efficiency of critical applications [5].

Over the past few years, *Micromachines MDPI* has published a wealth of innovative and groundbreaking research on fiber-optic sensors, highlighting their versatility and transformative potential across a wide range of applications. This Editorial brings attention to several notable contributions, showcasing advancements in the design, functionality, and implementation of fiber-optic sensor technology. By exploring these cutting-edge studies, the remarkable progress and diverse possibilities that fiber-optic sensors offer in fields such as biomedical diagnostics, structural health monitoring, and environmental sensing are underscored.

Recently, Chen et al. conducted an in-depth experimental investigation into the fabrication of fiber Bragg gratings (FBGs) using femtosecond (fs) laser point-by-point (PbP) inscription technology [10]. The study focused on analyzing the effects of key parameters, including focusing geometry, grating order, laser energy, and grating length, on the spectral characteristics of PbP FBGs. By optimizing these parameters, a high-quality first-order PbP FBG with exceptional performance was successfully fabricated, achieving a reflectivity of over 99.9% (corresponding to a Bragg resonance attenuation of 37.7 dB) and a remarkably low insertion loss of just 0.03 dB. Leveraging the versatility of fs laser PbP technology, high-quality FBGs were inscribed across a wide range of Bragg wavelengths, spanning from 856 nm to 1902.6 nm. Additionally, the rapid fabrication of wavelength-division-multiplexed (WDM) FBG arrays comprising 10 gratings was demonstrated. A Fabry–Perot cavity was also realized using two high-quality FBGs, and the birefringence of the cavity was significantly reduced—from 3.04 × 10^−5^ to 1.77 × 10^−6^—through a slit beam shaping-assisted fs laser PbP technique. These advancements underscore the potential of high-quality FBGs produced via fs laser PbP technology to enhance the performance of optical fiber sensors, lasers, and communication systems [10].

Graphene, a revolutionary two-dimensional carbon-based material with a hexagonal honeycomb lattice, zero-band gap, and exceptionally high specific surface area, possesses extraordinary optoelectronic properties, positioning it as a highly promising material for advanced optical fiber sensing applications [11]. Its integration into optical fiber sensors has attracted widespread attention across interdisciplinary domains such as biology, materials science, medicine, and micro-nano technologies. This surge in interest is driven by graphene’s remarkable attributes, including an ultra-high sensitivity, compactness, and robust resistance to electromagnetic interference, which make it ideal for challenging environments and precision sensing tasks [12].

Zhang et al. reviewed the latest advancements in graphene-based optical fiber biochemical sensors, emphasizing both the sophisticated fabrication methods for graphene materials and the intricate sensing mechanisms that enable their functionality [13]. Key developments in various sensor configurations—such as long-term fiber gratings, FBGs, no-core fibers, and photonic crystal fibers—are comprehensively examined, showcasing their unique capabilities and the performance enhancements facilitated by graphene. Furthermore, the future potential of graphene-enhanced optical fiber sensing technology was explored, outlining innovative opportunities for its application and development. These insights provide a critical foundation for driving progress in graphene-based optical fiber biochemical sensors, which are poised to redefine standards in precision sensing and multidisciplinary research applications [13].

In 2023, Lin et al. introduced an innovative fiber-optic sensor for 2D magnetic field sensing, leveraging the unique properties of the nanostructured magnetic fluid [14]. The sensor featured a ring-shaped fiber structure coated with magnetic fluid, whose distinctive magneto-optical characteristics enabled precise magnetic field detection. The interaction between the magnetic field and the nanostructured magnetic fluid was thoroughly analyzed using a 3D Monte Carlo simulation method, providing detailed insights into the induced magneto-optical changes. This sensor operated using intensity demodulation to achieve 2D vector magnetic sensing, demonstrating an impressive sensitivity of 2.402 dB/mT. The proposed design represents a significant advancement in high-sensitivity 2D vector magnetic field sensors, with promising applications in navigation systems, electrical power monitoring, and biological detection. By combining cutting-edge materials and advanced simulation techniques, this sensor sets a new benchmark for precision and functionality in magnetic field sensing technologies [14].

Fiber-optic Fabry–Perot pressure sensors have attracted significant attention in the in situ measurement of high-temperature pressures due to their compact size and exceptional anti-interference and anti-shock properties [15]. Despite these advantages, traditional designs often face challenges such as limited pressure measurement ranges, low sensitivity, and high fabrication complexity. To address these limitations, Duan et al. introduced a novel fiber-optic Fabry–Perot pressure sensor featuring a membrane–hole–base structure [16]. The sensitive core was fabricated using advanced laser cutting and direct bonding techniques to construct a robust three-layer sapphire design. Additionally, a dedicated large-cavity-length demodulation algorithm was developed to optimize the sensor’s Fabry–Perot cavity performance.

This innovative sensor design offered enhanced sensitivity, a streamlined structure, and simplified fabrication processes, while significantly improving pressure resistance and the ability to operate in harsh environments. The sensor demonstrated a broad pressure sensing range of 0–10 MPa within a temperature range of 20–370 °C, achieving a high sensitivity of 918.9 nm/MPa and a temperature coefficient of 0.0695 nm/(MPa∙°C). The measurement error remained within 2.312% across the entire temperature range. With its superior performance and robustness, this sensor is poised to advance applications in high-temperature pressure monitoring for industries such as aerospace, energy, and process engineering [16].

Yang et al. presented a novel air-gap fiber Bragg grating (g-FBG) sensor capable of simultaneous strain and temperature measurements [17]. The sensor was constructed by aligning two FBGs with an air gap between them, creating a unique structure that combines phase-shifted fiber Bragg grating (PSFBG) spectroscopy with Fabry–Perot interference (FPI) spectroscopy. This dual spectral approach leveraged the differing sensitivities of PSFBG and FPI spectra to strain and temperature, enabling the precise differentiation and measurement of these parameters. By analyzing the reflected dip in the PSFBG spectrum and the interference dip in the FPI spectrum, the sensor achieved a strain sensitivity of approximately 11.95 pm/με and a temperature sensitivity of about 9.64 pm/°C. The experimental results demonstrated a strain resolution of ±3.7 με within a range of 0 to 1000 με and a temperature resolution of ±0.6 °C within a range of 25 °C to 120 °C. This g-FBG sensor boasts a simple design, compact size, and cost-effective fabrication, making it a promising solution for multi-parameter sensing applications across diverse fields, including structural health monitoring, industrial process control, and environmental sensing [17].

Furthermore, Dong et al. presented a novel fiber optic microprobe displacement sensor, leveraging the unique properties of a micro-Michelson interference structure to achieve high-precision displacement measurements [18]. By addressing and eliminating the principal errors associated with micro-Fabry–Perot interferometric structures, the proposed sensor delivered superior accuracy and reliability. Two microprobe configurations—collimated and convergent—were analyzed, simulated, and designed to optimize their performance in distinct applications: long-distance displacement measurements and small-spot rough surface measurements, respectively. Key design parameters of the probes’ internal components were systematically mapped to coupling efficiency and measurement contrast, providing a comprehensive foundation for the optimization of probe performance. Simulation and experimental results demonstrated that the collimated probe achieves a working distance of up to 40 cm, while the convergent probe accommodates a tolerance angle of ±0.5°, making it suitable for a wide range of practical scenarios. These probes, when integrated into a fiber laser interferometer, enabled displacement measurements with an impressive resolution of 0.4 nm. This innovative sensor design offers a versatile and high-precision solution for application in fields such as precision manufacturing, metrology, and surface characterization [18].

In conclusion, we can say that fiber-optic sensors stand at the forefront of modern sensing technologies due to their unparalleled advantages, including high sensitivity, immunity to electromagnetic interference, compactness, and adaptability to harsh environments. Their versatile applications span across critical domains, such as structural health monitoring, medical diagnostics, aerospace, and telecommunications, showcasing their transformative impact. With the advancements in fabrication techniques, materials like graphene and innovations like Fabry–Perot cavities, fiber-optic sensors continue to push the boundaries of performance, enabling precise multi-parameter measurements. As the demand for robust, high-resolution, and multifunctional sensors grows, fiber-optic technology remains a dominant and indispensable tool for advances in science, industry, and technology.
